# Hyperoxia Induced Hypomyelination

**DOI:** 10.3390/biomedicines11010037

**Published:** 2022-12-23

**Authors:** Weilin Song, George Hoppe, Demiana Hanna, Tara M. DeSilva, Jonathan E. Sears

**Affiliations:** 1 Department of Ophthalmic Research, Cole Eye Institute, Cleveland Clinic, 9500 Euclid Avenue, Cleveland, OH 44195, USA; 2 Cleveland Clinic Lerner College of Medicine of Case Western Reserve University, 9500 Euclid Avenue, Cleveland, OH 44195, USA; 3 Department of Neurosciences, Lerner Research Institute, Cleveland Clinic, 9500 Euclid Avenue, Cleveland, OH 44195, USA; 4 Department of Cardiovascular and Metabolic Sciences, Lerner Research Institute, Cleveland Clinic, 9500 Euclid Avenue, Cleveland, OH 44195, USA

**Keywords:** periventricular leukomalacia, hyperoxia, hypoxia inducible factor, premature birth, oligodendrocytes, myelin basic protein, hypomyelination, prolyl hydroxylase, HIF stabilization

## Abstract

We asked whether hyperoxia might induce hypomyelination of the corpus callosum, clinically described as periventricular leukomalacia (PVL) of the severely preterm infant. Mouse pups and their nursing dams were placed in 80% oxygen from P4-P8, then removed to room air until P11. Corpus callosal sections were probed myelin immunofluorescence, tested for myelin basic protein concentration by Western blot, and both glial fibrillary acidic protein levels and apoptosis quantified. Density of corpus callosal capillaries were measured after lectin staining and hypoxia measured by Hypoxyprobe. Numbers of oligodendrocytes were quantified by immunohistochemistry. We next used hypoxiamimesis as a surrogate to hypoxia by comparing cerebral hypoxia inducible factor (HIF) stabilization to hepatic HIF stabilization. Hyperoxia induced hypomyelination and a reduction of corpus callosal capillaries. Hyperoxia decreased numbers of oligodendrocytes with an increase in corpus callosal fibrosis and apoptosis. Cerebral hypoxiamimesis induced hypomyelination whereas hepatic hypoxiamimesis alone increased myelination, oligodendrocyte numbers, and corpus callosal capillary density. Hepatic HIF-1 dependence on myelination was confirmed using the cre/lox hepatic HIF-1 knockout. These findings suggest that hyperoxia can induce hypomyelination through vasoobliteration and subsequent ischemia, adding a potential oxygen induced mechanism to the diverse causes of periventricular leukomalacia of the severely preterm infant. Targeting hepatic HIF-1 alone led to increased myelination.

## 1. Introduction

Premature birth, a major contributor to infant mortality worldwide, is associated with complications affecting a wide range of organ systems that have lifelong consequences for the health and development of premature infants [1]. These pathologies are increasing in incidence as more children are successfully resuscitated at lower birth weight and gestational age [2]. In particular, infants with very low birth weight (VLBW, complete list of abbreviations Table S1) or very preterm infants born before 32 weeks of gestation are at greater risk of developing diseases of prematurity, including periventricular leukomalacia (PVL) or brain white matter (WM) injury, retinopathy of prematurity (ROP), and bronchopulmonary dysplasia (BPD) [2].

PVL develops in up to 20% of VLBW infants and is associated with long-term cognitive and behavioral deficits in 25–50% of cases and with major motor deficits (e.g., cerebral palsy) in 10–15% of cases [3]. In addition, diffuse white matter injury is now more common than PVL [4,5]. PVL is characterized by deep WM necrotic lesions near the lateral ventricles and diffuse loss of pre-myelinating oligodendrocytes within the cerebral WM. The upstream triggers most commonly implicated in the pathogenesis of PVL are hypoxia/ischemia and intrauterine inflammation, although the exact mechanism by which these etiologies lead to WM injury have not yet been elucidated [6,7]. Animal models of PVL caused by exposure to global hypoxia and/or ischemia results in widespread white-matter injury and infarction, but do not recapitulate the periventricular focal lesions characteristic of the disease [8,9]. However, it should be emphasized that there are multiple phenotypes of abnormal myelination of the severely premature which perhaps suggest that different mechanisms shape these phenotypes [7].

Another proposed etiology of PVL is hyperoxia exposure from oxygen supplementation, which is required to prevent mortality in severely premature infants but simultaneously results in injury to still developing, premature tissues. In the case of ROP, a leading cause of childhood blindness worldwide, hyperoxia causes retinovascular growth attenuation through downregulation of hypoxia-inducible factor (HIF), a transcription factor that induces coordinated growth of blood vessels [10,11,12,13,14]. A decrease of supplemental oxygen as the infant matures after phase 1 retinal growth attenuation and vasoobliteration subsequently results in ischemia-driven pathologic neovascularization and retinal detachment [15,16]. The presence of excess oxygen in phase 1 causes the HIF prolyl hydroxylase domain protein (HIF PHD) to catalyze trans-4-prolyl hydroxylation of the alpha subunit of the HIF heterodimer at Pro-402 or Pro-564 within the C-terminal oxygen dependent degradation domain; the hydroxylated state makes it a substrate of the von Hippel Lindau protein (VHL), an E3 ubiquitin ligase, which polyubiquinates HIFα as a signal for proteasomal degradation [17,18,19,20,21,22]. Downregulation of HIF results in halted downstream angiogenic pathways, reduction of vascular endothelial growth factor (VEGF) and subsequent oxygen-induced retinovascular growth suppression and vascular obliteration [10,23]. Inhibition of HIF PHD during hyperoxia exposure with inhibitory analogues of α-ketoglutarate cofactor to HIF PHD, such as dimethyloxalylglycine (DMOG) or Roxadustat (FG-4592), prevents catabolism of HIF and was previously shown to induce protection against both oxygen-induced retinopathy (OIR) and BPD [24,25]. DMOG confers remote protection by targeting hepatic HIF-1 whereas Roxadustat promotes synergistic protection by targeting both the liver and the retina [25,26].

Given that ROP and PVL both affect infants within a critical gestational period and that the retina is a specialized extension of the central nervous system, we hypothesize that perhaps one of the many etiologies of PVL might parallel that of ROP, which is known to be caused by oxygen supplementation. Vascular imaging in human PVL infants reveals a pattern of deep WM vessel dropout similar to the vascular loss in ROP [27]. Immature, VEGF-dependent cerebral vessels are specifically susceptible to pathologic insult, and VEGF blockade within a specific gestational window leads to immature vessel regression and a PVL-like phenotype with focal lesions [28]. Additionally, HIF-mediated oxygen sensitivity within oligodendrocyte precursor cells (OPCs) has been shown to play a critical role in coordinating postnatal WM vascular development and myelination in the developing brain. HIF stabilization within oligodendrocytes maintains their precursor form and inhibits myelination, which can occur only after oligodendrocytes reach maturity [29]. Therefore, either hypoxic preconditioning through biphasic oxygen parameters or pharmacologic preconditioning through early HIF stabilization as a strategy to ameliorate oxygen induced pathologies of the severely preterm infant requires the determination of how systemic HIF stabilization, shown beneficial to preventing ROP and BPD, affects CNS myelination. In murine models, hyperoxia has been shown to cause WM injury and disrupt the maturation of OPCs, but little is reported on the effects of hyperoxia on WM vasculature within a model of oxygen-induced brain injury [30,31].

In this study, we develop a model of hyperoxia-induced brain injury and demonstrate that hyperoxia exposure results in loss of cerebral WM vessels and subsequent proximal ischemia. Our findings further suggest that both the timing and location of HIF stabilization plays a role in the pathogenesis and protection against PVL through preservation of capillaries within the WM to promote downstream myelination by reducing oxygen induced.

## 2. Materials and Methods

### 2.1. Animals

The Cleveland Clinic Institutional Animal Care and Use Committee (protocol no. 2019-2183) approved protocols for live mice. Wild-type C57BL/6J mice (stock 664), Gt(ROSA)26Sor^TM1(Luc)Kael^ (Luc-ODD) transgenic mice (stock 6206) expressing HIF-1α oxygen-dependent degradation domain fused to luciferase were purchased from Jackson Laboratory, Bar harbor, ME, USA. The conditional liver HIF-1α KO mouse was created at Jackson Laboratory by crossing a HIF-1α^2lox/2lox^ mouse (stock 7561) with an Albumin-Cre mouse (stock 3574) and has been previously validated [26].

### 2.2. Oxygen-Induced Brain Injury Model

Two litters of pups born on the same day were cross-fostered starting on postnatal day 1 (P1). Half of the pups from both litters were placed with a lactating mother into 80% oxygen from P4 to P8, while the other half remained in room air with the second lactating mother. A Plexiglas incubator with an oxygen sensor and feedback system (ProOx, Biospherix, Parish, NY, USA) was used to ensure continuous hyperoxia. The mothers were switched every 24 h until P8 to minimize oxygen-induced acute lung injury. Half of the mouse pups were sacrificed (a described below) at P8, immediately after removal from the 80% oxygen chamber, and the other half at P11. Brains and retinas were dissected out for immunohistochemistry and Western blot analysis. The hyperoxia pups appeared normal and did not suffer weight loss (control: P8, 3.93 ± 0.122 g; P11, 5.10 ± 0.11 g; hyperoxia: P8, 3.76 ± 0.16 g; P11, 4.88 ± 0.24 g, n ≥ 6) during or after the hyperoxia exposure. Hyperoxia protocol was performed with C57BL/6 as well as Luc-ODD and liver HIF-1α KO mice pups.

### 2.3. Injection Protocol with Prolyl Hydroxylase Inhibitors

Stock concentrations of 20 mg/mL DMOG (Frontier Scientific, Logan, UT, USA) were made by dissolving compound in sterile phosphate-buffered saline (PBS). These solutions were filter sterilized and stored in aliquots at −80 °C. 50 mg/mL stock concentrations of Roxadustat (FG-4592, AdooQ Bioscience, Irvine, CA, USA) were made by dissolving compound in dimethyl sulfoxide, then further diluted in sterile PBS to 1 mg/mL and stored aliquoted at −80 °C. All HIF prolyl hydroxylase inhibitors were injected intraperitoneal (IP) using a 31-gauge needle at a dose of 200 mg/kg for DMOG and 10 mg/kg for Roxadustat.

Injection intervals were adapted from the protocol used for the OIR model, and involved three IP injections of either DMOG or Roxadustat at P3, P5, and P7 in the oxygen-induced brain injury model reported above. Equivalent volume of PBS was injected in control mice.

### 2.4. Brain Immunohistochemistry

Mice were anesthetized with isoflurane (Piramal Critical Care, Bethlehem, PA, USA) and transcardial perfused with cold PBS and fresh 4% paraformaldehyde (PFA) at P8 and P11. Brains were dissected out and post-fixed with 4% PFA overnight at 4 °C, and cryopreserved in a 30% sucrose, 0.05% sodium azide solution at 4 °C for 48 h. Tissues were then embedded in tissue freezing medium (Tissue-Tek O.C.T. Embedding Compound, Electron Microscopy Sciences, Hatfield, PA, USA) and serially cut into coronal sections (30 µm) by a Leica CM1950 Cryostat (Leica Biosystems). Free floating tissue sections were washed with PBS to remove O.C.T. compound and blocked and permeabilized with 0.3% Triton-X, 1% BSA, and 5% goat serum in PBS for 1 h at room temperature. Sections were then incubated with primary antibody overnight at 4 °C. The following primary antibodies and their dilutions were used: MBP (1:1000, SMI-99, Biolegend, San Diego, CA, USA), Olig2 (1:300, AB9610, Sigma-Aldrich, St. Louis, MO, USA), GFAP (1:250, GA5, Invitrogen Life Technologies, San Diego, CA, USA), CC1 (1:300, AB16794, Abcam, Boston, MA, USA), Activated-Caspase 3 (1:250, C92-605, BD Pharmingen, San Diego, CA, USA), as well as Isolectin GS-IB4 Alexa Fluor 568 Conjugate (1:100, Invitrogen Life Technologies, San Diego, CA, USA).

Three washes with PBS were performed at room temperature. Fluorophore-conjugated secondary antibody (donkey anti-mouse IgG Alexa Fluor 488, goat anti-rabbit IgG Alexa Fluor 488, donkey anti-mouse Alexa Fluor 594, donkey anti-rabbit IgG Alexa Fluor 594, 1:1000, Invitrogen, San Diego, CA, USA) was applied and incubated for 2 h at room temperature followed by three washes with PBS. Sections were mounted onto glass slides with anti-fade mounting medium (VectaShield, Vector Labs, Newark, CA, USA) with or without DAPI (4′,6-diamidino-2-phenylindole).

### 2.5. Brain Tissue Hypoxia Analysis

For detection of tissue hypoxia with the Hypoxyprobe Green Kit (NPI Inc., Burlington, MA, USA), pups were given a single IP injection of pimonidazole HCl (60 mg/kg) dissolved in PBS either at P8 or P11. Pups were sacrificed and brains dissected 90 min after injection. P8 mice were sacrificed six hours after removal from 80% oxygen. Following tissue processing for immunofluorescent staining as detailed above, brain sections were incubated with FITC-conjugated mouse monoclonal anti-pimonidazole adduct antibody (1:100 dilution) overnight before mounting.

### 2.6. Brain Microscopy and Immunohistochemistry Measurements

Following immunofluorescent staining, brain sections were imaged with fluorescent microscope (Zeiss Axio Imager.Z1, Carl Zeiss, White Plains, NY, USA). Three different laser lines were used: DAPI (400 nm excitation, 470 nm emission filter), FITC (488 nm excitation; 522/35 emission filter), and CY3 (560 nm excitation; 605/32 emission filter). Images quantified by mean fraction area of fluorescence (MBP, GFAP, Hypoxyprobe) were obtained using 5× or 10× objective on a single plane and analyzed by the ImageJ (NIH, Bethesda, MD, USA) software as previously described [32]. Exposure and threshold parameters were consistent across images within each experiment during imaging and analysis. Brain vasculature was imaged with Zeiss ApoTome 3 at 10× objective for improved optical resolution. Images were quantified using mean vessel area by the AngioTool (NIH, Bethesda, MD, USA) software [33]. Images quantified by cell number/volume (Olig2, CC1, Activated Casp-3) were imaged as z-stacks using 20× objective and depth of 20 μm. Cells were counted by analyzing the merged image for each z-stack on ImageJ and identifying positive immunofluorescence for each individual channel. Measurements from 3–5 images of external capsule (EC), corpus callosum, or subventricular zone (SVZ) from different tissue sections were taken and averaged for each animal analyzed.

### 2.7. Western Blotting

Protein analysis was performed on brain hemispheres after removing olfactory bulbs and brainstem. Tissue was lysed in 4 °C radioimmunoprecipitation assay (RIPA) buffer solution (Sigma-Aldrich, St. Louis, MO, USA) containing protease inhibitor cocktail Complete (Roche, Mannheim, Germany), homogenized using a tight-fitting microtube pestle, sonicated, and centrifuged at 20,000× *g*, 4 °C for 15 min to remove particulate matter. A Pierce BCA protein assay kit (Thermo-Fisher, Waltham, MA, USA) was used to measure protein concentrations using spectrophotometry at 562 nm. Proteins samples (16 μg per lane) were resolved on gradient 4–20% polyacrylamide Tris-glycine gels (Life Technologies, San Diego, CA, USA) and electro-transferred to polyvinylidene difluoride membrane (Millipore, Danvers, MA, USA) for immunoblotting. Membranes were blocked with Intercept blocking buffer (LI-COR Biosciences, Lincoln, NE, USA), then probed with primary antibodies overnight: MBP (1:1000, SMI-99, Biolegend, San Diego, CA, USA), HIF-1α (1:500, Cayman Chemical, Ann Arbor, MI, USA), and β-actin (1:5000, Cell Signaling Technology, Danvers, MA, USA). Membranes were then washed and incubated in secondary fluorophore-conjugated antibody (described above) at room temperature for 1 h before fluorescence imaging with LI-COR Odyssey CLx and quantification with Image Studio (LI-COR Biosciences, Lincoln, NE, USA). Densitometry measurements for proteins of interest were normalized to β-actin.

### 2.8. Detection of Luc-ODD Luciferase Reporter In Vitro

HIF stabilization levels were assessed in the Luc-ODD mouse strain by measuring luciferase activity [34]. Luc-ODD mice were placed in hyperoxia from P4–P8 and anesthetized and transcardial perfused by PBS at P8 and P11 without fixation in PFA. Following brain extractions, the WM (corpus callosum) was micro-dissected out and placed in Glo Lysis Buffer (Promega, Madison, WI, USA), homogenized with disposable microtubule pestles, followed by centrifugation at 20,000× *g*, 4 °C for 15 min. A BCA protein assay (Thermo-Fisher, Waltham, MA, USA) was used to measure protein concentrations of tissue preparations following dilution with Glo Lysis Buffer in a total volume of 100 μL into white opaque 96-well plates. Room temperature extracts were mixed with 100 uL of luciferase substrate Bright-Glo Luciferase Assay System (Promega, Madison, WI, USA); the Victor X2 (PerkinElmer, Waltham, MA, USA) luminometer/fluorimeter was used to measure luminescence.

### 2.9. Reverse Transcription and Quantitative PCR

Brain tissue (whole brain) was placed into 1 mL of RNAlater reagent (Qiagen, Germantown, MD, USA) and stored at 4 °C. Total RNA was extracted using the RNeasy kit (Qiagen, Germantown, MD, USA) and measured using standard spectrophotometric parameters on NanoDrop (Thermo-Fisher, Waltham, MA, USA). RNA (1 μg) from each sample was retrotranscribed to cDNA using High-Capacity cDNA Reverse Transcription Kit (Applied Biosystems, Waltham, MA, USA). cDNA samples (2 μL) were used as the template for amplification reactions performed with the TaqMan Fast Advanced Master Mix and Vegfa or Epo TaqMan assay FAM and Hprt TaqMan assay VIC in a QuantStudio 3 Real-Time PCR System (all from Applied Biosystems, Waltham, MA, USA). Quantitative PCR data analysis was performed with QuantStudio Design and Analysis software v1.4.3 (Applied Biosystems, Waltham, MA, USA).

### 2.10. Statistical Analysis

Means were compared using Student’s and Mann-Whitney *t*-test. The unpaired two-tailed probability associated with rejecting the null hypothesis of no difference between observed groups was calculated, using an alpha level of 0.05. Error bars in all figures represent standard error of the mean (SEM). An online software was used and samples were analyzed in a double-blind fashion [35].

## 3. Results

### 3.1. Hyperoxia Results in Decreased Myelination in the Developing Mouse Brain

**Figure 1:**
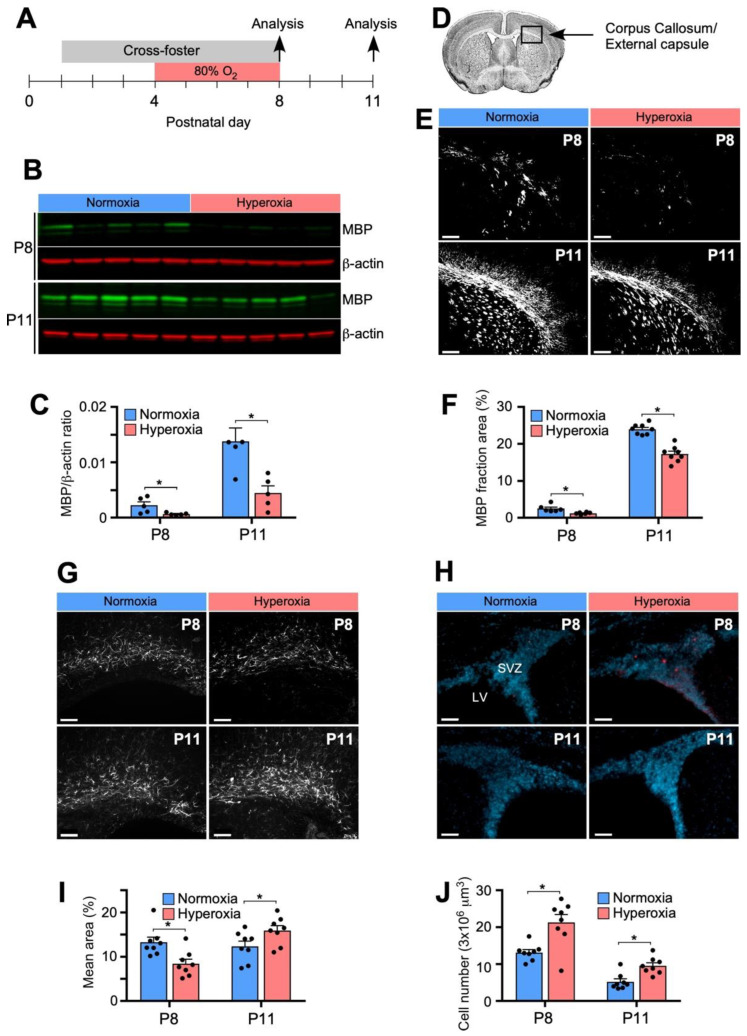
Hyperoxia induced model of periventricular leukomalacia (PVL). (**A**) Schema for two C57Bl/6J mice litters that were cross-fostered from P1; half of the litters were exposed to hyperoxia (80% oxygen) and half remained in normoxia (room air) from P4-P8. (**B**) Western blots for MBP and β-actin at P8 and P11. (**C**) Densitometric analysis of MBP normalized to β-actin showed decreased protein expression at P8 and P11. (**D**) Region of the Corpus Callosum/External Capsule imaged for immunohistochemical staining. (**E**) Representative fluorescent images (5×) of MBP immunohistochemical staining at P8 and P11. Consistent exposure and threshold parameters between groups were used during imaging and analysis. Scale bar = 200 μm. (**F**) Quantification of myelin basic protein (MBP) immunofluorescence as mean percent area showed decreased MBP staining at P8 and P11. (**G**) Hyperoxia is associated with altered GFAP immunoreactivity and cellular apoptosis within the SVZ. Representative fluorescent images (20×) of GFAP-stained brain sections at P8 and P11 in hyperoxia and normoxia brains. Scale bar = 50 μm. (**I**) Quantification of GFAP immunofluorescence as mean percent area showed decreased immunoreactivity at P8 and increased at P11. (**H**) Representative images (20×) of SVZ stained with activated Casp-3 (red) and DAPI at P8 and P11. Scale bar = 50 μm. (**J**) Quantification of activated Casp-3+ cells within the SVZ within hyperoxia brains at both time points. For A-F, data is shown as mean ± SEM for each group ((**A**–**F**), n = 4–6 animals, 5 images/animal). For G-J, each group and time point, data is shown as mean ± SEM and n = 5–7 brains. An unpaired two-tailed *t* test was used to compare normoxia vs. hyperoxia, where * *p* < 0.05, was used for statistical analysis. (MBP = myelin basic protein, SVZ = supraventricular zone, LV = lateral ventricle, GFAP = glial fluorescent acidic protein).

Myelin basic protein (MBP) is one of the major proteins in myelin and its expression levels directly correlates with progression of developmental myelination [36]. We therefore examined the effect of hyperoxia on MBP protein levels as a marker for WM development by immunofluorescence and Western blot analyses. For this study, two litters born on the same day were cross-fostered from P1 to P8 to ensure normoxia controls were litter-matched and to reduce oxygen-induced damage in the nursing dams. Half of the pups in each litter were exposed to 80% oxygen from P4 to P8, which roughly corresponds to gestational weeks 24–37 in humans in regard to CNS development (Figure 1A). Compared to normoxia litter-matched controls, mice exposed to hyperoxia had marked decrease in MBP protein expression on Western blot analysis at P8, measured 3 h after removal from hyperoxia, that persisted after 3 days in normoxia at P11 (Figure 1B,C). Immunofluorescent staining of brain sections also revealed decreased areas of MBP immunofluorescence in the external capsule (EC; Schema Figure 1D) at P8 and P11 in mice exposed to hyperoxia compared to controls (Figure 1E,F).

### 3.2. Hyperoxia Is Associated with Reactive Astrogliosis and Increased Apoptosis in the Developing Brain

In addition to diffuse abnormalities in myelination and a loss of pre-myelinating OPCs, PVL presents clinically with astrogliosis and focal periventricular lesions [37]. We found that hyperoxia followed by normoxia created a biphasic response in WM astrocytes, demonstrated by staining of glial fibrillary acidic protein (GFAP), following 80% oxygen exposure from P4 to P8 (Figure 1G,I). Immediately after hyperoxia at P8 there was decreased GFAP immunoreactivity in hyperoxia brains compared to normoxia controls. In contrast, at P11 the hyperoxia brains had greater GFAP immunoreactivity within the EC and hypertrophy of astrocytic processes.

We next assessed for signs of cellular apoptosis by staining for activated caspase-3 (Casp-3), the main effector caspase of the apoptotic cascade within cells [38]. Although apoptosis within the subventricular zone (SVZ) occurs in normal brain development during neurogenesis, we found a greater number of activated Casp-3+ cells within the SVZ in P8 and P11 brains following hyperoxia exposure compared to litter-matched normoxia controls (Figure 1H,J).

### 3.3. Hyperoxia Results in Decreased Capillary Density in the Retina and Brain

Within the developing rodent brain, there is massive proliferation of capillary beds through generation of new angiogenic sprouts during the first two weeks after birth [39]. After confirming that our model of hyperoxia exposure from P4 to P8 results in alterations to myelination within the developing WM, we next investigated the effects of hyperoxia on developing vasculature within the brain and retina.

**Figure 2:**
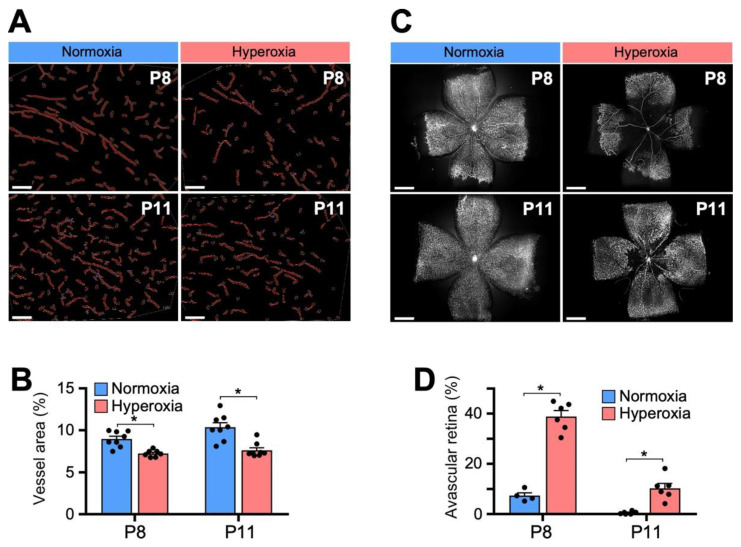
Hyperoxia decreases capillary network in the brain and retina. (**A**) Vasculature segmentation with AngioTool of representative fluorescent images (10×) of lectin immunohistochemical staining of brain capillary networks supplying the external capsule at P8 and P11 in normoxia versus hyperoxia. Scale bar = 100 μm. (**B**) Brain capillary networks supplying the EC were quantified as mean vessel area in each image. There was decreased vessel area following hyperoxia at both P8 and P11. (**C**) Representative images of lectin-stained retina flat mounts at P8 and P11 after 80% oxygen exposure from P4–P8. Each image was obtained by merging 16 overlapping fluorescent images. Scale bar = 200 μm. (**D**) Quantification of retinal flat mounts depicting fraction avascular area of total retinal area. There was increased avascular fraction area at P8 and P11 following hyperoxia. Data shown as mean ± SEM for each group. For each group and time point, 5 images/animal were averaged for 5–8 animals. An unpaired two-tailed *t* test comparing normoxia vs. hyperoxia, where * *p* < 0.05, was used for statistical analysis.

Staining with fluorophore-conjugated lectin of brain sections was analyzed double blinded using AngioTool software to quantify the vascular network and expressed as vessel area. There was a decrease in vasculature supplying the EC (Figure 2A,B) and periventricular zones (not shown) at both P8 and P11 following hyperoxia exposure.

The effects of hyperoxia on capillaries supplying the WM paralleled the effects seen in the retina, demonstrating the susceptibility of immature microvasculature within the central nervous system to high oxygen states (Figure 2C,D). In the most commonly used mouse model of OIR, exposure to 75% oxygen from P7–P12 consistently resulted in vasoobliteration of retinal capillaries and subsequent hypoxia-induced neovascularization [40]. We found that in our model of oxygen-induced brain injury, 80% oxygen exposure from P4 to P8 similarly led to marked capillary dropout and increased avascular areas in lectin-stained retinal flat mounts immediately after hyperoxia at P8 that persisted after 3 days of room air at P11.

**Figure 3:**
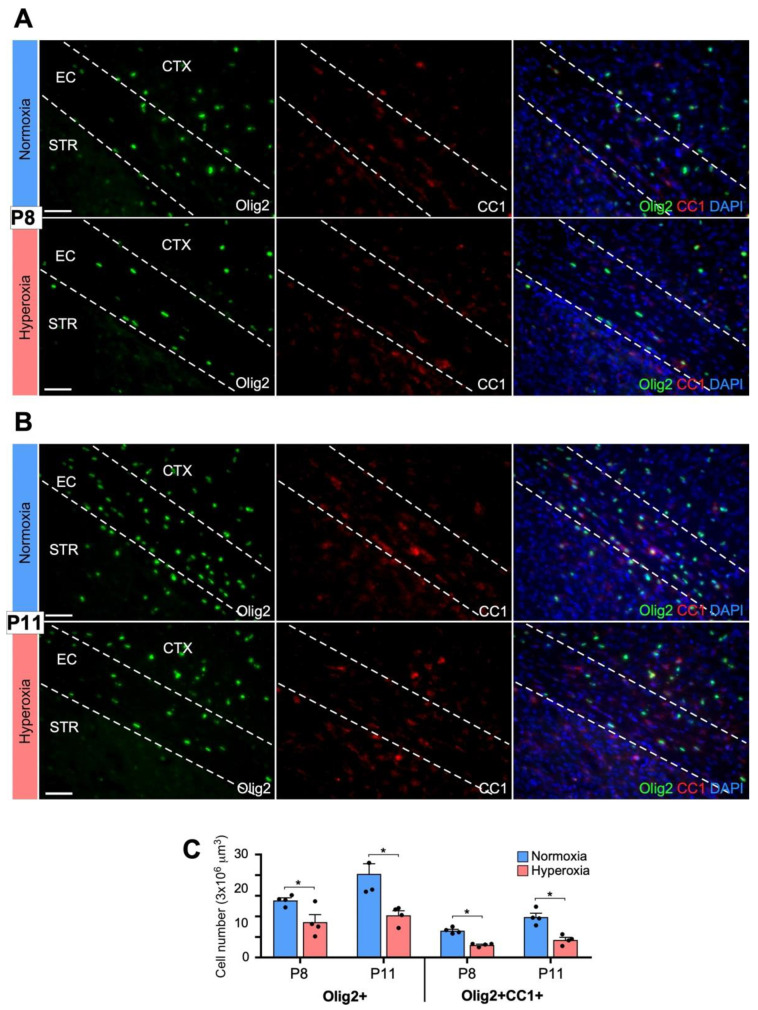
Hyperoxia exposure results in reduction of Olig2+ and CC1+ oligodendrocytes within immature white matter. EC, external capsule; CTX, cortex; STR, striatum. Olig2, marker of all oligodendrocytes, CC1, marker of mature oligodendrocytes. Dashed lines depict the margins of the EC. (**A**) Fluorescent images at P8 (20×) showing reduction in the number of Olig2+ (green) and Olig2+CC1+ (green and red) oligodendrocytes in hyperoxia vs. normoxia. Scale bar = 50 μm. (**B**) The reduction of oligodendrocytes at P8 is sustained at P11 within the EC, marked by dashed white lines. Scale bar = 50 μm. (**C**) Quantification of Olig2+ and Olig2+CC1+ cells within the EC at P8 and P11 in normoxia and hyperoxia. Data shown as mean ± SEM for each group (n = 4–6 animals, 5 images/animal), using an unpaired two-tailed *t* test comparing normoxia vs. hyperoxia; * *p* < 0.05. Using non-parametric Mann–Whitney analyses, (**C**) (Olig+2 at P8), is not statistically significant.

We next examined the cellular changes underlying the observed hyperoxia-induced decrease in MBP expression in the developing WM. The progression of the oligodendrocyte lineage from OPCs to mature myelinating oligodendrocytes has been well characterized [36]. We found that following hyperoxia exposure from P4 to P8, mice pups had a reduction in the number of Olig2+ cells (total oligodendrocytes) and Olig2+/CC1+ cells (mature oligodendrocytes) at both P8 and P11 within the EC (Figure 3A–C). To avoid including off-target staining of other cell types, only cells that stained for both Olig2 and CC1 were counted as mature oligodendrocytes. Although statistically significant by parametric analysis, using a non-parametric Mann–Whitney analyses, Olig+2 cells at P8, were not statistically significant (Students *t*-test, *p* = 0.033; Mann–Whitney, *p* = 0.057).

### 3.4. Relative Hypoxia in Developing Brain after Removal from Hyperoxia

**Figure 4:**
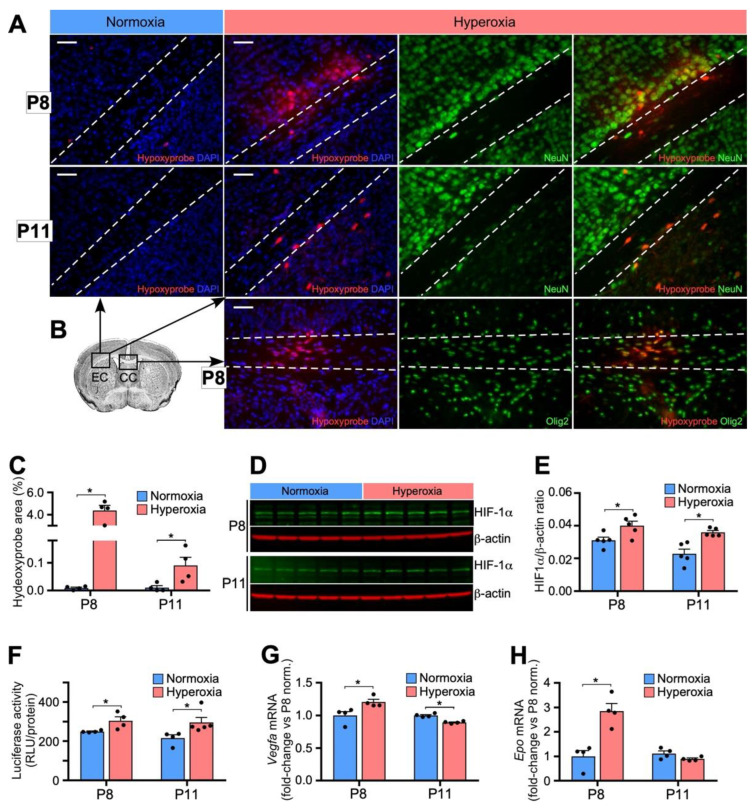
Hyperoxia induces relative hypoxia. (**A**) Hyperoxia from P4–P8 increases Hypoxyprobe staining compared to normoxia at P8, 6 h after removal from hyperoxia, and at P11, 3 days after removal from hyperoxia. Double-staining for either neurons (green, NeuN) upper 2 panels, oligodendrocytes (green, Olig2) and red (Hypoxyprobe) reveals both hypoxic neurons and oligodendrocytes after phase 1 hyperoxia followed by phase 2 relative hypoxia. Dashed lines mark the histological margins of the EC or CC as shown in the following schematic. Scale bar = 50 μm. (**B**) Schema demonstrating area of sections from either the Corpus Callosum (CC) or the External Capsule (EC). (**C**) Quantification of Hypoxyprobe at P8 and P11 compared to normoxia. (**D**) Western blot and (**E**) quantification of Western blot probed with HIF-1α antibody shows statically significant upregulation of HIF-1α in brain tissue 3 h after removal from hyperoxia at P8 and 3 days after removal from hyperoxia at P11. (**F**) The luciferase-oxygen dependent degradation domain transgenic mouse confirms a downregulation of HIF prolyl hydroxylase activity (reflected as an increase in luciferase protein) equivalent to an increase in HIF stability. (**G**) mRNA regulated by HIF, VEGF and (**H**) erythropoietin are increased at P8, 3 h after removal from hyperoxia. n = 4 for all groups except Western blot, where n = 5. Data is shown as mean ± SEΜ for each group; an unpaired two-tailed *t* test was used to compare normoxia vs. hyperoxia, where * *p* < 0.05, was used for statistical analysis. Using non-parametric Mann–Whitney analyses, (**E**) (HIF-1a at P8) is not statistically significant.

We then wanted to assess whether there were physiologic consequences from the reduction in capillary network observed following hyperoxia exposure. P8 mice were sacrificed six hours after removal from hyperoxia. Both P8 and P11 mice were injected with pimonidazole HCl (60 mg/kg) 90 min before being sacrificed. Subsequent immunofluorescent staining of brain sections of pimonidazole adducts showed hypoxic tissue at P8 and P11 in the brains of mice exposed to hyperoxia, but no evidence of hypoxia in the normoxia controls (Figure 4A–C). At P8, there were concentrated areas of hypoxic cells located throughout the brain tissue, including proximal to and within WM tracts (Figure 4A–C) and throughout the striatum and cortex (not shown). After co-staining with NeuN, a marker for neurons, and Olig2, a marker for oligodendrocytes, we found that the areas of hypoxic cells proximal to the WM tracts were hypoxic neurons, while the areas of hypoxic cells within the WM tracts, including the corpus callosum, were hypoxic oligodendrocytes (Figure 4A,B). At P11, there were individual hypoxic cells located throughout the brain tissue rather than in concentrated areas that were identified as neurons (Figure 4A,B).

We next examined the molecular changes correlating with the hypoxia observed in brain tissue following removal from hyperoxia. We first performed a Western blot to measure protein levels of HIF-1α in the brain at P8, six hours after removal from hyperoxia, and at P11. Within the developing brain, HIF-1/2 was shown to have the dual effect of promoting angiogenesis and inhibiting maturation of oligodendrocytes. We found increased HIF-1α levels following hyperoxia exposure at both time points, that is, P8, 6 h after removal from hyperoxia, and P11, 3 days after removal from hyperoxia (Figure 4D,E). Although statistically significant by parametric analysis, using a non-parametric Mann–Whitney analyses, HIF-1a levels at P8 were not statistically significant (Students *t*-test, *p* = 0.029; Mann–Whitney, *p* = 0.055).We emphasize that in samples labeled hyperoxia, measurements were made in relative hypoxia, that is after hyperoxic treatment caused vasoobliteration and pups were in normoxia (relative hypoxia) for 6 h (P8) and 3 days (P11). We next used the luciferase-oxygen dependent degradation domain mouse (Luc-ODD) to show where HIF is upregulated. In this mouse, luciferase expression matches HIF stabilization because luciferase is fused to the C-terminal regulatory element that post-translationally targets HIF-1α to the proteasome if the ODD is hydroxylated under conditions of hyperoxia. However, in hypoxia, no hydroxylation occurs and therefore the luciferase fusion protein is spared and luminescence correlates to HIF stabilization [34]. Following hyperoxia exposure from P4–P8 and then removal to normoxia in Luc-ODD mice, brain luciferase activity was increased at both P8 (6 h after removal from hyperoxia) and P11 (3 days after removal from hyperoxia, Figure 4F), supporting the finding that HIF-1α was upregulated in the developing brain after removal from hyperoxia.

We evaluated mRNA levels of two direct downstream targets of HIF-1α, VEGF and erythropoietin, and found that there were increases in both *Vegfa* and *Epo* mRNA at P8 six hours after removal from hyperoxia (Figure 4G,H).

### 3.5. Systemic HIF-Stabilization during Hyperoxia ALTERS Myelination and oligodendrocyte Populations in the Developing Brain 

In order to test the paradigm that oxygen induced vasoobliteration which led to hypoxia, we next examined the effect of directly stabilizing HIF in the CNS, i.e., inducing the molecular outcome of hypoxia, known as hypoxiamimesis. Pharmacologic HIF-stabilization through administration of the HIF PHD-inhibitors DMOG and Roxadustat (FG-4592) in the classic OIR model was previously shown to reduce OIR and BPD in an oxygen induced retinal and lung disease model. Although both compounds inhibit the HIF PHD by binding the 2-oxoglutarate cofactor binding site, DMOG is completely catabolized within blood to MOG, and cleared by hepatic monocarboxylate transporter 2 (MCT2) so that HIF in peripheral tissues is not stabilized by intraperitoneal DMOG [41]. This makes hepatic HIFa a selective target of DMOG [25,26,42]. Roxadustat, however, can be found in CNS tissues as well as multiple other organs [25]. This liver specific tropism of DMOG versus multi-organ targeting of Roxadustat has been definitively demonstrated by Western blot, Luc-ODD transgene analysis, transcriptome studies of liver and retina from animals treated with either DMOG or Roxadustat, previous experiments analyzing hepatic HIF-1a ablation, and liquid chromatography mass spectrometry (LCMS) of plasma and retina [23,25,26,41,42]. Given the capillary dropout we observed in the vasculature supplying the WM following hyperoxia exposure, which paralleled the vasoobliteration within the retina, we wanted to examine the effects on WM myelination and vasculature after simulating hypoxia chemically during the hyperoxia exposure using the hypoxiamimetics Roxadustat versus DMOG to assess whether oxygen might affect myelination through HIF.

**Figure 5:**
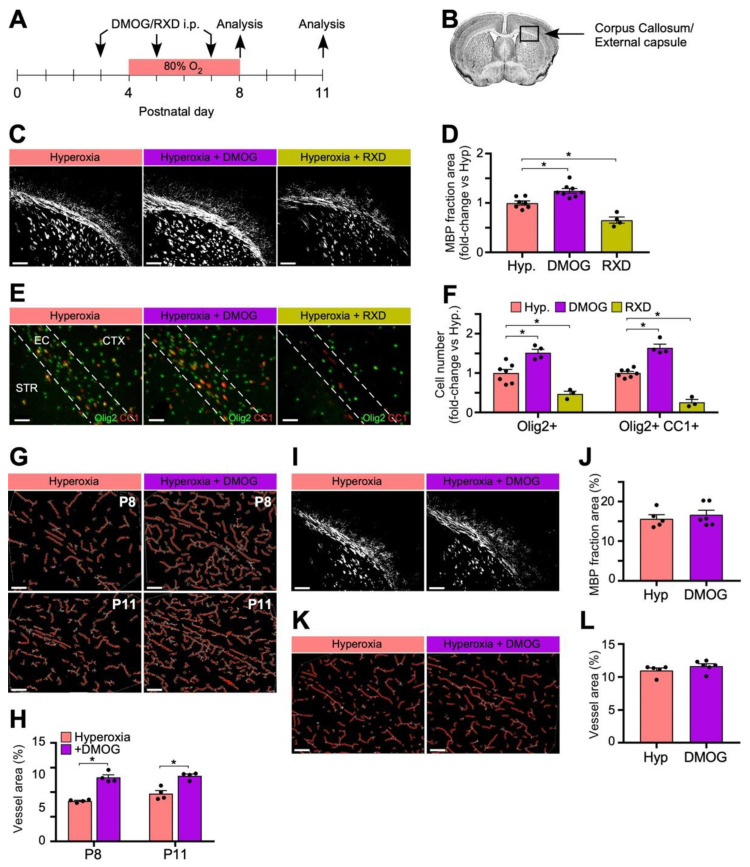
Systemic HIF-stabilization decreases and hepatic HIF stabilization increases myelination, oligodendrocyte populations, and vascular density at P11. (**A**) Schema demonstrating hyperoxia and HIF stabilization protocols. Mice were injected IP with DMOG (200 mg/kg) or Roxadustat (RXD) (10 mg/kg) at P3, P5, and P7 and placed in 80% oxygen from P4 to P8. (**B**) Areas that were imaged in the Corpus Callosum/External capsule. (**C**) Representative fluorescent images (5×) of myelin basic protein (MBP) immunohistochemical staining at each time point after applying consistent exposure and threshold parameters between groups during imaging and analysis. Scale bar = 200 μm. (**D**) Quantification of MBP immunofluorescence as mean percent area normalized to hyperoxia shows increased MBP staining after DMOG and decreased MBP staining after Roxadustat, imaged at P11. (**E**) Fluorescent images (20×) showed increased Olig2+ (green) and Olig2+CC1+ (green and red) oligodendrocytes after DMOG and decreased numbers after Roxadustat in the corpus callosum. Scale bar = 50 μm. (**F**) Quantification of Olig2+ and Olig2+CC1+ at P11. (**G**) Representative images of brain capillary networks supplying the EC stained with lectin and analyzed by AngioTool. Scale bar = 100 μm. (**H**) Quantification of mean vessel area demonstrates increases following hyperoxia at both P8 and P11 in the DMOG group. (**I**) Representative images of immunofluorescent MBP in the corpus callosum shows no difference in the hepatic HIF-1 KO. Scale bar = 200 μm. (**J**) Quantification of MBP comparing DMOG treatment in the hepatic HIF-1 KO. (**K**) Representative brain capillary images from the hepatic HIF-1 KO with and without DMOG treatment. Scale bar = 100 μm. (**L**) Quantification of brain capillary density shows no effect of DMOG in the hepatic HIF-1 KO. No difference in MBP in the hepatic HIF-1 KO demonstrates that DMOG induced protection is dependent on hepatic HIF-1. Data is shown as mean ± SEM for each group at P11 unless indicated otherwise (n = 4–6 animals, 4 images/animal), using an unpaired two-tailed *t* test comparing drug vs. hyperoxia litter-matched control; * *p* < 0.05. (Hyp = hyperoxia, DMOG = dimethyloxalylglycine, RXD = Roxadustat).

In mice pups exposed to 80% oxygen from P4 to P8, we injected half of the litter intraperitoneally with either Roxadustat (10 mg/kg) or DMOG (200 mg/kg) at P3, P5, and P7 (Schema, Figure 5A). Litter-matched controls were injected with an equal volume of PBS. Roxadustat decreased MBP immunofluorescence compared to litter-matched hyperoxia controls at P11 in samples from the corpus callosum and external capsule (Figure 5B–D), but liver trophic DMOG conversely increased MBP immunofluorescence (Figure 5C,D).

Staining for Olig2 and CC1 within the EC at P11 subsequently revealed an increase in numbers of Olig2+ and Olig2+CC1+ cells following DMOG administration compared to litter-matched hyperoxia controls (Figure 5E,F), consistent with an increase in myelination. After injections with Roxadustat, however, there was a decrease in the number of Olig2+ and Olig2+CC1+ cells within the EC compared to hyperoxia controls (Figure 5E,F) consistent with a previous report that conditional KO of oligodendrocyte VHL (constitutive stabilization of HIF-1) inhibits OPC differentiation and myelination [29]. Furthermore, while there was no difference in the fraction of mature to total oligodendrocytes between the DMOG and control groups, there was a 50% reduction in the fraction of mature to total oligodendrocytes in the Roxadustat group compared to control. This suggests that in addition to reducing the number of total oligodendrocytes, administration of Roxadustat also inhibited progression of oligodendrocyte precursors and immature oligodendrocytes into mature, myelinating oligodendrocytes. The overall differences observed in oligodendrocyte populations were consistent with and may explain the differences in MBP expression between the two HIF-stabilizing drugs: DMOG confers remote protection by stabilizing hepatic HIF alone whereas Roxadustat globally stabilizes HIF in extra-visceral organs such as the eye and the brain [25]. This finding indirectly supports our hypothesis that hyperoxia induces capillary loss that secondarily upregulates HIF and downregulates myelination. The fact that Roxadustat mimicked hypoxia even in hyperoxia, and decreased myelination, is a compelling correlation to the finding that oligodendrocyte HIF stabilization decreased progression to mature myelin producing cells [7,29]. This is indirect support for the hypothesis that vasoobliteration could induce hypoxia and upregulate HIF in oligodendrocytes.

### 3.6. Remote HIF-Stabilization during Hyperoxia Promotes Brain Vasculature

We have definitively demonstrated that DMOG is catabolized in liver alone and does not stabilize extrahepatic HIF-1 yet protects retinal vasculature from oxygen induced retinopathy [25,26,42]. We assessed the effects of DMOG administration on retina and brain vasculature following hyperoxia exposure at P8 and P11. Within the brain, DMOG administration was associated with an increase in vasculature network supplying the EC at both P8 and P11 compared to hyperoxia controls (Figure 5G,H).

### 3.7. Remote HIF-Stabilization Has No Effects in Myelination and Vasculature in Liver-Specific HIF-1 KO Mice

In order to confirm that the changes in WM myelination and vasculature seen after DMOG administration were dependent on stabilization of HIF-1 within the liver, and not from an off-target effect of DMOG, we repeated the above experiment in liver HIF-1-KO mice (HIF-1α^2lox/2lox^; albumin-cre) [26], and found no increase in MBP immunofluorescence between mice who received DMOG and hyperoxia litter-matched controls as we saw in wild type mice (Figure 5I,J). We also found no difference in the vasculature network supplying the WM between DMOG treated and untreated hepatic HIF-1 KO mice (Figure 5K,L). These experiments confirm that the DMOG induced protective phenotype requires hepatic HIF-1.

## 4. Discussion

**Figure 6:**
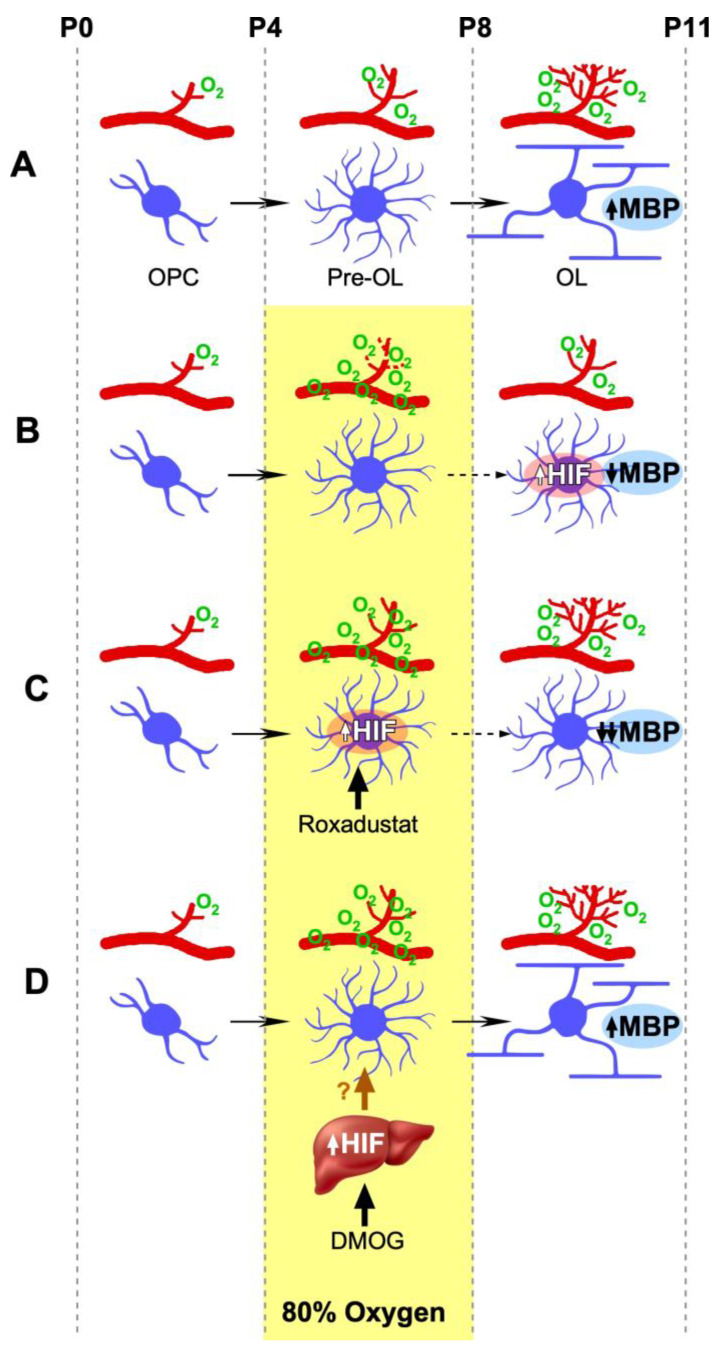
Two-step hypothesis of the effect of oxygen on myelination. (**A**) Normoxia allows myelination by inducing normal capillary growth at postnatal day 4-postnatal day 8 (P4–P8) to prevent hypoxia. Normal vascularization, in turn, downregulates HIF in premature oligodendrocytes (pre-OLs) allowing their transformation to mature myelinating oligodendrocytes (OLs). Myelination proceeds normally. (**B**) Excess oxygen early in postnatal development, P4–P8, causes loss of capillaries and subsequent hypoxia that leads to HIF stabilization in pre-OLs, preventing their maturation into myelinating OLs. Myelination is decreased. (**C**) Roxadustat, a systemic HIF stabilizer that enters the central nervous system, stabilizes HIF in pre-OLs, preventing their maturation into myelinating OLs. Myelination is decreased. (**D**) DMOG, administered intraperitoneally during P4–P8, is a liver trophic HIF stabilizer that does not enter the CNS, yet increases brain capillary density even in hyperoxia at P4–P8, allowing for normal myelination, as there is no ischemia and no hypoxia to upregulate HIF after P8 in pre-OLs. Pre-OLs mature into myelinating OLs. Myelination proceeds normally. (P = postnatal day, OPC = oligodendrocyte precursors, Pre-OL = premature olig9dendrocytes, OL = mature myelinating oligodendrocytes, HIF = hypoxia inducible factor, MBP = myelin basic protein, DMOG = dimethyloxalylglycine, ? = yet unknown liver-derived HIF-activated factors that confer remote protection).

During normal development, postnatal myelination and angiogenesis are reported to be coupled by HIF-1/2, which has the dual effect of inhibiting OPC maturation and promoting angiogenesis [29]. The timing and degree of HIF activity is critical as physiologic hypoxia in utero must promote tissue vascularization to prevent hypoxia without stabilizing HIF in oligodendrocytes, which decreases myelination [29]. This dual mechanism helps ensure that myelination only proceeds when blood supply is sufficient to meet the metabolic demands of oligodendrocytes (see schematic Figure 6). Therefore, hyperoxia exposure during a period of robust CNS angiogenesis, which in a rodent is the first postnatal week [39], may disrupt proper angiogenesis of immature vasculature resulting in insufficient blood supply to the white matter. Subsequent proximal ischemia and upregulation of HIF-1/2 following removal from hyperoxia (into relative hypoxia) may lead to impaired myelination secondary to inhibition of OPC maturation. This is the model proposed by Yuen et al. which our data supports [7].

In this study, we investigated a murine model of oxygen-induced WM injury that parallels one of the many varied clinical presentations of hypomyelination in premature infants. We found that after 80% oxygen exposure from P4 to P8, mice pups had reduced myelination and number of total and mature oligodendrocytes within the developing white matter, as well as increased periventricular reactive astrogliosis and cellular apoptosis. The initial decrease followed by an increase in GFAP expression was also observed in hippocampal astrocytes in a model of transient global ischemia, and may reflect astrocyte dysfunction and reactivity secondary to fluctuations in oxygen levels [43]. In a previously reported model of hyperoxia-induced premature brain injury, MBP expression was similarly decreased immediately after hyperoxia from P6 to P8 and persisted until P12. While they found that MBP expression returned to control levels by P15, MRI imaging revealed long-term WM damage in P30 and P60 mice as well as impairment in motor coordination and activity consistent with PVL [31,44]. Our findings support the possibility that one cause of white matter loss associated with premature birth can be hyperoxia.

The decreased myelination observed following hyperoxia exposure is likely due to the reduced number of mature oligodendrocytes within the white matter at P8 and P11. While previous studies point to the increased susceptibility of pre-myelinating oligodendrocytes to hyperoxia-induced apoptosis [30,31], this would not fully explain the persistent decrease we observed at P11. The vasculature dropout we observed following hyperoxia could be contributing to the reduction in oligodendrocyte populations in two ways. First, following removal from hyperoxia, there were marked hypoxic regions within the white matter that we identified as Olig2+ cells at P8. We further found upregulation of HIF-1α hours after removal from hyperoxia at P8 that persisted until P11, which is consistent with the hypothesis that HIF-1/2 upregulation may be inhibiting maturation of OPCs within a relative hypoxic environment caused by hyperoxia-induced vasoobliteration. Second, because OPCs require vasculature as a physical scaffold when migrating, hyperoxia-induced capillary dropout may also lead to decreased oligodendrocyte numbers within the white matter by impairing their migration [45].

While DMOG and Roxadustat are both HIF-stabilizing agents that promote protection against OIR during hyperoxia, their opposing effects on myelination is likely attributed to differences in their pharmacokinetics. Roxadustat targets a number of organs, including the liver, retina, and brain [25,46]. Direct HIF-stabilization within the brain with Roxadustat resulted in exacerbation of hypomyelination and reduced oligodendrocyte numbers compared to hyperoxia controls, supporting the previously reported finding that HIF-stabilization by disruption of oligodendrocyte pVHL (HIF upregulation) results in inhibition of oligodendrocyte maturation [29]. In contrast, DMOG solely targets the liver and was previously shown to not be protective against OIR in liver HIF-1 KO mice, demonstrating that DMOG-dependent protection of retinal vasculature occurs remotely and requires stabilization of hepatic HIF-1α [26]. We found in this study that in addition to protection of retinal vasculature and lung tissue, administering DMOG during hyperoxia was associated with increased cerebral vasculature, myelination and number of myelinating oligodendrocytes within the white matter compared to hyperoxia alone. OPCs were previously shown to require vasculature as a physical scaffold for migration throughout the central nervous system from progenitor domains, and the increased vasculature network secondary to DMOG administration may promote OPC migration to the WM and contribute to the increased numbers of oligodendrocytes observed [45]. As would thus be expected, administration of DMOG in liver HIF-KO (HIF-1α^2lox/2lox^; albumin-cre) mice did not affect white matter myelination or vasculature following hyperoxia exposure.

All together, these results further support the idea that oxygen-induced toxicity is a systemic condition, unifying the pathogenesis of ROP/BPD/PVL, and that the liver could be stimulated to protect remote organ tissues from oxygen-induced injury [26,47]. While the exact mechanisms by which hepatic HIF-stabilization remotely protects the retina and other peripheral organs remain to be fully elucidated, others and we have definitively demonstrated that HIF-1 dependent activation of serine/1-carbon metabolism is a critical metabolic pathway in adaptation to hypoxia and is required in the remote protection against OIR following HIF-stabilization in the liver [48]. In addition, serine also serves as a primary building block for the glycosphingolipid biosynthesis required to produce myelin, and taurine, a product of serine/1-carbon metabolism, supplementation was shown to promote oligodendrocyte maturation through increasing the serine pool and promoting flux through the glycosphingolipid biosynthetic pathways [49]. Our previous experiments demonstrated that metabolites of serine/1-carbon metabolism were decreased in the hepatic HIF-KO in both plasma and retina even with DMOG induced hypoxiamimesis, suggesting that in the mouse pup, retinal serine is derived from hepatic serine biosynthesis and could explain the dependence of CNS myelination on the liver [42].

In conclusion, our findings suggest the possibility that pathogenesis of ROP/BPD/PVL can be similarly dependent on hyperoxia. Hypoxic preconditioning of severely premature infants or low dose, intermittent hepatic hypoxiamimesis through liver trophic small molecule HIF PHD inhibition (HIF PHi) may be constructive strategies to protect the severely premature infant.

## 5. Conclusions

One Mechanism of CNS hypomyelination, in addition hypoxia/ischemia, inflammation, and intraventricular hemorrhage, may be hyperoxia alone. Hyperoxia can decrease blood vessel density in white matter and reduce overall oligodendrocyte numbers. Hepatic HIF-1 stabilization can increase corpus callosal myelination. Further work Is necessary to define the mechanism of remote protection by the liver.

## Data Availability

Data is contained within the article.

## Supplementary Materials

The following supporting information can be downloaded at: https://www.mdpi.com/article/10.3390/biomedicines11010037/s1, Table S1, Abbreviations.
