# Supplementary material for: Hyperoxia Induced Hypomyelination
Source: Biomedicines. 2022 Dec 23;11(1):37. doi: 10.3390/biomedicines11010037 (PMC9855863; doi:10.3390/biomedicines11010037)
Supplement: Supplementary file 1 [file biomedicines-11-00037-s001.zip › biomedicines-2089953-supplementary.pdf]

Table S1. Abbreviations

Abbreviation	Description
BPD	Bronchopulmonary dysplasia
Casp-3+	Activated caspase 3
CC	Corpus callosum
CC1	Staining for mature oligodendrocytes
CNS	Central nervous system
CTX	Cortex
DAPI	4’,6-diamidino-2-phenylindole
DMOG	Dimethylglycine
DMSO	Dimethyl sulfoxide
EC	External capsule
FG	Roxadustat
GFAP	Glial fibrillary acidic protein
GSL	Glycosphingolipid
HIF	Hypoxia-inducible factor
IHC	Immunohistochemistry
IP	Intraperitoneal
Luc-ODD	Transgenic mouse with luciferase fused to oxygen-degradation domain of HIFα
MBP	Myelin basic protein
NeuN	Staining for neurons
OIR	Oxygen induced retinopathy
Olig2	Staining for all oligodendrocytes
OPC	Oligodendrocyte precursor cell
P4	Postnatal day 4

PBS	Phosphate-buffered saline
PFA	Paraformaldehyde
PHD	Prolyl hydroxylase domain protein
PVL	Periventricular leukomalacia
RIPA	Radioimmunoprecipitation assay
ROP	Retinopathy of prematurity
SEM	Standard error of the mean
STR	Striatum
SVZ	Subventricular zone
VEGF	Vascular endothelial growth factor
VLBW	Very low birth weight
WM	White matter
WT	Wild type
